# Hepatitis B virus X protein disrupts the balance of the expression of circadian rhythm genes in hepatocellular carcinoma

**DOI:** 10.3892/ol.2014.2570

**Published:** 2014-09-26

**Authors:** SHENG-LI YANG, CHAO YU, JIAN-XIN JIANG, LI-PING LIU, XIEFAN FANG, CHAO WU

**Affiliations:** 1 Department of General Surgery, Liyuan Hospital, Tongji Medical College, Huazhong University of Science and Technology, Wuhan, Hubei 430077, P.R. China; 2 Department of Hepatobiliary Surgery, Affiliated Hospital of Guiyang Medical College, Guiyang, Guizhou 550001, P.R. China; 3 Department of Hepatobiliary and Pancreatic Surgery, Shenzhen People’s Hospital, Second Clinical Medical College, Jinan University, Shenzhen, Guangdong 518000, P.R. China; 4 Department of Pediatrics, College of Medicine, University of Florida, Gainesville, FL 32610, USA

**Keywords:** hepatitis B virus X protein, circadian clock, hepatocellular carcinoma

## Abstract

The human circadian rhythm is controlled by at least eight circadian clock genes and disruption of the circadian rhythm is associated with cancer development. The present study aims to elucidate the association between the expression of circadian clock genes and the development of hepatocellular carcinoma (HCC), and also to reveal whether the hepatitis B virus X protein (HBx) is the major regulator that contributes to the disturbance of circadian clock gene expression. The mRNA levels of circadian clock genes in 30 HCC and the paired peritumoral tissues were determined by reverse transcription-quantitative polymerase chain reaction (RT-qPCR). A stable HBx-expressing cell line, Bel-7404-HBx, was established through transfection of HBx plasmids. The mRNA level of circadian clock genes was also detected by RT-qPCR in these cells. Compared with the paired peritumoral tissues, the mRNA levels of the *Per1*, *Per2*, *Per3* and *Cry2* genes in HCC tissue were significantly lower (P<0.05), while no significant difference was observed in the expression levels of *CLOCK*, *BMAL1*, *Cry1* and *casein kinase 1ɛ* (*CK1ɛ*; P>0.05). Compared with Bel-7404 cells, the mRNA levels of the *CLOCK*, *Per1* and *Per2* genes in Bel-7404-HBx cells were significantly increased, while the mRNA levels of the *BMAL1*, *Per3*, *Cry1*, *Cry2* and *CKIɛ* genes were decreased (P<0.05). Thus, the present study identified that disturbance of the expression of circadian clock genes is common in HCC. HBx disrupts the expression of circadian clock genes and may, therefore, induce the development of HCC.

## Introduction

Hepatocellular carcinoma (HCC) is the fifth most common type of cancer and the third leading cause of cancer-related mortality worldwide (1,2). Of the risk factors attributed to liver carcinogenesis, chronic hepatitis B virus (HBV) infection has been demonstrated as a major factor (3). The double-stranded DNA genome of HBV contains four overlapping open-reading frames that encode the surface protein, the core protein, a polymerase and the HBV X protein (HBx) (4). HBx is a multifunctional protein that does not bind directly to DNA, but exerts transcriptional activation through its interaction with nuclear transcription factors and the modulation of cytoplasmic signal transduction pathways, including NF-κB signaling (5). HBx has been demonstrated to accelerate the progress of HCC in numerous processes, including apoptosis, proliferation, inflammation, angiogenesis, immune responses, multi-drug resistance, invasion and metastasis (6). Thus, there is increasing evidence demonstrating the crucial role of HBx in the development and progression of HCC.

The circadian clock is the inner rhythm of organisms, which has been formed over the course of long-term evolution and it regulates daily rhythmic variations in various physiological processes, including sleep and activity, appetite, hormone levels, metabolism and gene expression (7,8). The pacemakers are located in the suprachiasmatic nucleus (SCN) of the hypothalamus and produce self-sustaining circadian rhythms that are synchronized by external cues (9). Circadian rhythms similar to those operating in the SCN have been found in the majority of mammalian cells and peripheral tissues. These peripheral circadian rhythms can be driven or synchronized by the central pacemaker in the SCN through neuronal and humoral factors (10). In humans, the molecular clocks that these intrinsic rhythmic changes are based upon are the transcription-translation feedback loops of multiple biological clock genes, including *CLOCK*, *BMAL1*, *Per* (*Per1*, *Per2* and *Per3*), *Cry* (*Cry1* and *Cry2*) and *casein kinase 1ɛ* (*CKIɛ*) (11).

The *CLOCK-BMAL1* heterodimer initiates transcription by binding E-boxes, which are CACGTG nucleotide sequences in promoters, and drives the rhythmic transcription of the three *Per* and two *Cry* genes. As *Per* and *Cry* are translated in the cytoplasm, they form *Per-Cry* complexes and translocate into the nucleus to inhibit further *CLOCK-BMAL1* transcriptional activity (12). In addition, *CKIɛ* has also been found to be involved in the transcriptional loops by controlling the stability of the *PER* and *BMAL1* proteins via phosphorylation (13).

On the basis of epidemiological and experimental studies, the potential association between disruption of the circadian rhythm and tumor development has become a focus of investigation (14,15). The disturbance of circadian clock gene expression is common in colorectal (16), breast (17) and pancreatic cancers (18), and is generally associated with the development and progression of these types of tumor. The disturbance of circadian clock gene expression has also been found in HCC (19), however, the predominant factors that cause the circadian clock disorder have not yet been elucidated. The present study aimed to reveal the role of HBx in contributing to the disturbance of the expression of circadian clock genes in HCC.

## Materials and methods

### Tumor specimens and cell lines

Tissues were obtained from 30 HCC patients who underwent surgical resection at the Department of Hepatobiliary Surgery, Affiliated Hospital of Guiyang Medical College (Guiyang, China) between October 2010 and June 2011. All the tissue samples were obtained from the patients prior to any medical treatment. All patients tested positive for the HBV surface antigen, HBsAg, and negative for antibodies to the hepatitis C virus (anti-HCV) and human immunodeficiency virus (anti-HIV). The mean patient age was 44.9 years (range, 23–62 years), with 26 male and four female patients. Clinical data, tumor characteristics, and American Joint Committee on Cancer staging of these patients are demonstrated in Table I (20). All specimens were obtained between 9:00 a.m. and 12:00 p.m. on the same day. The viable tumor and adjacent healthy tissues were dissected immediately and snap-frozen in liquid nitrogen. The human HCC BEL-7404 cell line was obtained from the American Type Culture Collection (Manassas, VA, USA) and cultured in Eagle’s Minimum Essential Medium (Sigma-Aldrich, St. Louis, MO, USA), supplemented with 10% fetal bovine serum. The BEL-7404 cells were transfected with pEGFP-HBx and pEGFP empty vectors, and incubated in a selection media containing 800 mg/l G418 (Gen-View Scientific Inc., Calimesa, CA, USA) for 14 days. The BEL-7404 cells transfected with pEGFP empty vectors served as the control. Stable colonies were isolated and identified by reverse transcription-polymerase chain reaction (RT-PCR) and western blot analysis.

### RNA extraction and first-strand complementary (c)DNA synthesis

Fresh frozen tissues (weight, 150–200 mg) were used to isolate total RNA with 1 ml TRIzol^®^ reagent (Invitrogen Life Technologies, Carlsbad, CA, USA). The quantity and quality of the extracted RNA was determined using a NanoDrop Spectrophotometer (ND-2000; NanoDrop Technology, Wilmington, DE, USA). The cDNA synthesis was performed at 42°C for 60 min in a 25-μl volume, which contained 2 μg RNA, 1.6 μM Oligo(dT)18, 0.6 μM deoxyribonucleotides, 200 units Moloney murine leukemia virus reverse transcriptase (Promega, Madison, WI, USA) and the reaction buffer that was supplied.

### qPCR

Following RT, the cDNA samples were diluted in RNAse-free water (ratio, 1:5). The primer sequences used in the current study are presented in Table II. Each reaction used the SYBR^®^ Green mix (Invitrogen Life Technologies), a cDNA template, 10 μM forward primer, 10 μM reverse primer and double-distilled H_2_O in a total volume of 20 μl, and was performed on the ABI 7500 Real-Time PCR System (Applied Biosystems Life Technologies, Foster City, CA, USA). The reaction conditions were as follows: Denaturation at 94°C for 60 sec, 40 cycles of annealing at 55°C for 60 sec and extension at 72°C for 60 sec. The program was set to automatically record the average fluorescence value of the last 10% of time in the final cycle, which was equal to the quantity of amplification at the end of each cycle. Following completion of the reactions, the baseline and threshold were adjusted on the ABI 7500 Real-Time PCR System software, where the cycle threshold (Ct) value of each reaction well was read. The data were analyzed according to the comparative Ct method and normalized according to the GAPDH expression in each sample. The primers were designed according to the cDNA sequences in the GenBank database (National Center for Biotechnology Information, Bethesda, MD, USA) using Primer Express software (Applied Biosystems Life Technologies) and are listed in Table II. The qPCR was performed in triplicate.

### Protein preparation and western blot analyses

Cells were collected and lysed in lysis buffer containing 50 mmol/l Tris-HCl (pH 8.5), 150 mmol/l NaCl, 0.2 g/l NaN_3_, 0.1 g/l SDS, 100 μg/ml phenylmethylsulfonyl fluoride, 1 μg/ml aprotinin, 10 ml/l NP-40, and 5 g/l sodium deoxycholate. Cells were centrifuged at 20,800 × g for 15 min to remove the cellular debris. The protein concentrations were determined by the Bradford method. A total of 30–50 μg of protein was separated by sodium dodecyl sulfate (SDS)-polyacrylamide gel electrophoresis using 12% SDS polyacrylamide gels, transferred to polyvinylidene fluoride membranes, blocked in 5% non-fat milk in Tris-buffered saline containing 0.1% Tween-20 for 2 h, incubated with primary mouse monoclonal antibodies raised against baculovirus expressed recombinant Hep B xAg (obtained from Santa Cruz Biotechnology, Inc. (sc-57760, Dallas, TX, USA) for HBx (1:400), and β-actin raised against gizzard actin (1:2,000; sc-47778) for 1 h at 37°C and incubated overnight at 4°C, followed by a 1-h incubation with the appropriate horse-radish peroxidase conjugated monoclonal secondary goat anti-mouse IgG antibody. The bands were visualized using an enhanced chemiluminescence (ECL) detection system (Thermo Fisher Scientific Inc., Rockford, IL, USA).

### Statistical analysis

Data were expressed as means ± standard deviation and were analyzed using SPSS version 13.0 (SPSS Inc., Chicago, IL, USA). The gene expression levels in the liver cancer tissues were compared with those of the adjacent normal tissues using the Wilcoxon test. In addition, the differences between the Bel-7404-HBx and control cells were analyzed using the Mann-Whitney U test and P<0.05 was considered to indicate a statistically significant difference.

## Results

### Disturbance of the expression of circadian clock genes is commonly identified in HCC

**Figure 1:**
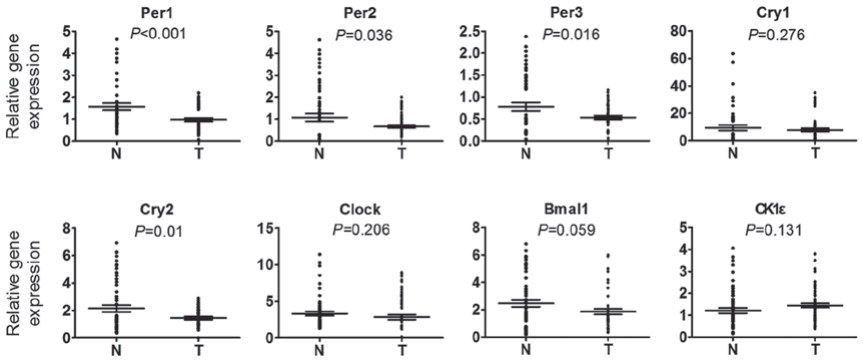
Comparison of circadian gene mRNA expression levels between hepatocellular carcinoma (HCC) tissue (T) and matched peritumoral tissue (N). Box boundaries represent the 25th and 75th percentiles of the observed values; capped bars, the 10th and 90th percentiles; solid line, the median. P-values were calculated by the Wilcoxon test. *Per1*, *Per2*, *Per3* and *Cry2* gene expression levels were higher in the peritumoral tissue than in the HCC tissue. *CLOCK*, *BMAL1*, *Cry1* and *CK1ɛ* gene expression levels were similar in the HCC and peritumoral tissue. P<0.05 was considered to indicate a statistically significant difference.

The mRNA levels of circadian clock genes were first determined in 30 HCC and the paired peritumorous tissues using RT-qPCR. It was found that the mRNA levels of *Per1*, *Per2*, *Per3* and *Cry2* in HCC tissues were markedly decreased in comparison with those in the paired peritumoral tissues, while no significant difference was observed in the mRNA levels of *CLOCK*, *BMAL1*, *Cry1* and *CK1ɛ* compared with the peritumoral tissues (Fig. 1). In these cases, the majority (70–90%) of the HCC cancerous tissues exhibited downregulation of the circadian clock gene, whereas the remaining cases were either downregulated in the non-cancerous tissues or were cases in which no differential expression of the circadian clock genes was detected. In particular, *Per1* and *Per3* downregulation was found in 27 (90%) out of the 30 cases analyzed and only three cases (10%) exhibited *Per1* and *Per2* upregulation in the HCC tissues. It was also found that the majority of the HCC tissues exhibited downregulation of at least four circadian clock genes. Therefore, it could be inferred that the disturbance of circadian clock gene expression is a common event in HCC and results in the disruption of normal circadian rhythm in cancerous cells.

### HBx causes disturbance of circadian clock gene expression in HCC cells

**Figure 2:**
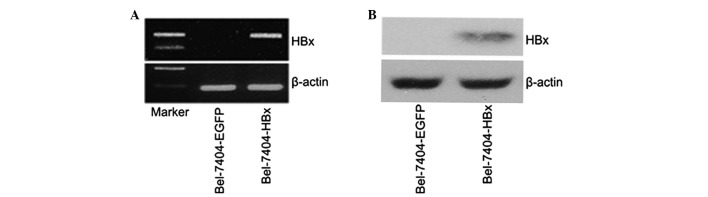
Identification of stable HBx transfection in Bel-7404 cells. (A) Integration of the HBx gene into the engineered cells was identified by reverse-transcription polymerase chain reaction using genomic DNA as a template; β-actin served as a loading control. (B) Western blot analysis reveals the expression of HBx in Bel-7404 cells. HBx, hepatitis B virus X protein.

**Figure 3:**
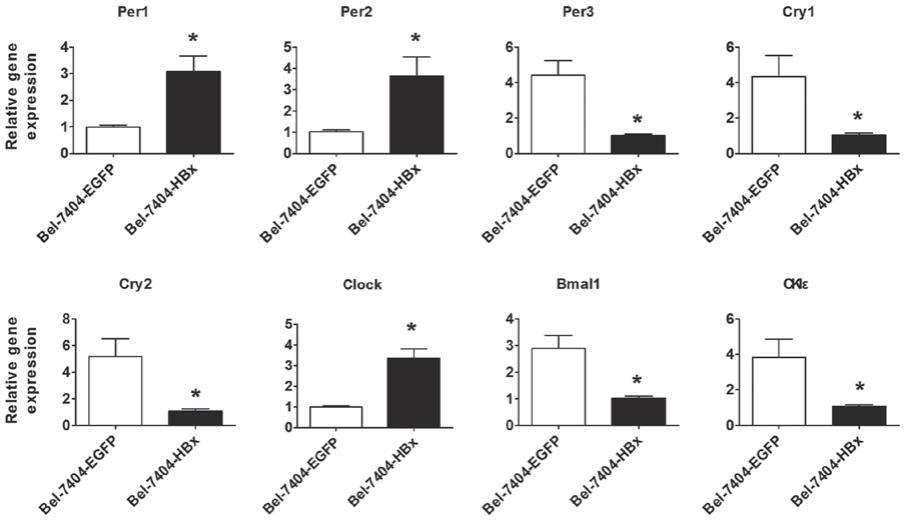
Comparison of circadian gene mRNA expression levels between the Bel-7404-HBx and control cells. The expression of *CLOCK*, *Per1* and *Per2* mRNA was increased in the Bel-7404-HBx cells compared with the control cells, while the expression of *BMAL1*, *Per3*, *Cry1*, *Cry2* and *CKIɛ* mRNA was decreased in the Bel-7404-HBx cells. ^*^P<0.05 vs. BEL-7404-EGFP.

HBx is a multifunctional protein that plays a vital role in the development and progression of HCC. To identify the influence of HBx on the expression of circadian genes, a stable HBx-transfected Bel-7404 cell line was established, termed Bel-7404-HBx cells. Subsequently, HBx mRNA and protein expression was identified in the HBx-transfected cells. As shown in Fig. 2A, HBx mRNA was highly expressed in the Bel-7404-HBx cells, with no HBx mRNA detected in the control cells. In addition, western blot analysis detected the 17-kDa HBx protein in the lysates of the Bel-7404-HBx cells (Fig. 2B). Therefore, HBx was successfully transfected and expressed in the Bel-7404-HBx cells. The expression of circadian clock genes in these cells was assessed. Compared with the control cells, the expression of *CLOCK*, *Per1* and *Per2* mRNA was significantly increased in the Bel-7404-HBx cells, while the expression of *BMAL1*, *Per3*, *Cry1*, *Cry2* and *CKIɛ* mRNA was significantly decreased (Fig. 3; P<0.05). Therefore, HBx may disturb circadian clock gene expression in HCC cells.

## Discussion

An increasing number of studies have demonstrated that breast, colon and prostate cancers are more common among individuals whose circadian rhythms are constantly disturbed as a result of shift work, jet lag or increased exposure to light at night (16,21,22). Further studies have revealed that the disturbance of circadian clock gene expression is particularly common in various malignancies (15,23,24), and that circadian clocks guide a number of cancer-related genes, including genes that regulate cell division, DNA repair and apoptosis (25,26). Therefore, the disruption of circadian clock genes and their downstream clock-controlled genes may enhance cancer development, and this viewpoint has already been demonstrated in murine cancer models (27,28).

The association between circadian clock genes and HCC has not been fully elucidated. The present study has found that the mRNA levels of *Per1*, *Per2*, *Per3* and *Cry2* in HCC cancerous tissues were significantly lowered compared with the paired peritumoral tissues, while no significant difference was observed in the expression levels of *CLOCK*, *BMAL1*, *Cry1* and *CK1ɛ*. This finding was consistent with the study by Lin *et al* (19), which revealed that the expression of *Per1*, *Per2*, *Per3* and *Cry2* was lower in HCC tissue samples. The expression of these genes was also found to be lower in colorectal and pancreatic cancer (18,29). However, it was demonstrated that there was a significant correlation between the low levels of *Per1*, *Per2*, *Per3*, and *Cry2* and poor patient survival rates, implying that these genes may be closely associated with the carcinogenesis and development of abdominal cancer (18,29).

The *Per* genes have been studied extensively and it has been proposed that these genes act as tumor suppressors, with prior reports indicating that decreased *Per* gene expression is associated with tumorigenesis and disease progression (30,31). The molecular link between *Per* genes and cell cycle control incorporates their inhibitory effect on cyclin D1, c-Myc and Wee1, leading to either a repressed G_1_-S transition or an enhanced G_2_-M transition (32,33). *Per1* and *Per2* are involved in the DNA damage response signaling pathways, perhaps as cofactors with checkpoint kinase 2 (Chk2) for the activation of ataxia telangiectasia mutated (ATM). In murine models, *Per2* mutations result in temporal changes in mRNA expression for genes associated with cell cycle regulation and tumor suppression, including c-Myc, cyclins and mouse double minute 2 homolog, resulting in an impaired DNA damage response and accelerated tumor growth (34,35). *Per3* interacts with ATM and Chk2. In addition, silencing of *Per3* expression terminates Chk2 activation upon induction of DNA damage as *Per3* overexpression alone activates Chk2, resulting in decreased cellular proliferation and apoptosis (36).

Although circadian clock genes were found to be the most prevalent, abnormally expressed genes in the tumor cells the underlying mechanisms of circadian rhythm disorders remain unclear. The present study reveals that HBx may alter the expression of the circadian clock genes at the level of mRNA in HCC cell lines. The expression of *CLOCK*, *Per1*, and *Per2* mRNA was upregulated, while the expression of *BMAL1*, *Per3*, *Cry1*, *Cry2* and *CKIɛ* mRNA was downregulated. This observation may be an alternative cause of HBx-induced HCC carcinogenesis and development. Therefore, a comparison between the alteration trends of HCC tissues and HBx-expressing HCC cells was made. It was found that only *Per3* and *Cry2* were downregulated in the two cell lines; therefore, HBx disturbs the expression of circadian clock genes, however, it may not be the major regulator in HCC.

Further investigation is required to fully elucidate the influence of HBx on the circadian clock, and its association with carcinogenesis and development of HCC. In addition, more studies are necessary to obtain an improved understanding of the association between circadian rhythm disruption and HCC, which may broaden current knowledge on the occurrence and development of HCC (37). This association may provide novel approaches and methods for the treatment of HCC.

## Figures and Tables

**Table I:** Clinicopathological features of 30 patients with hepatocellular carcinoma.

Clinicopathological parameter	Cases, n (%)
Mean age (range)	45 years (23–62 years)
Gender	
Male	26 (86.7)
Female	4 (13.3)
Cirrhosis	
Presence	17 (56.7)
Absence	13 (43.3)
Tumor size	
<3 cm	11 (36.7)
≥3 cm	19 (63.3)
Vascular invasion	
Presence	5 (16.7)
Absence	25 (83.3)
Tumor number	
1	25 (83.3)
>1	5 (16.7)
Tumor differentiation	
Well	5 (16.7)
Moderate	19 (63.3)
Poor	6 (20.0)
AJCC stage	
I	16 (53.3)
II	8 (26.7)
III	6 (20.0)

AJCC, American Joint Committee on Cancer (20).

**Table II:** Polymerase chain reaction primers and conditions.

Gene	Primer	Temperature (°C)	Product size (bp)
*Per1*	5′-AGGCAACGGCAAGGACTC-3′ 5′-GGCTGTAGGCAATGGAACTG-3′	60.2	101
*Per2*	5′-CTACAGCAGCACCATCGTC-3′ 5′-CCACTCGCAGCATCTTCC-3′	58.9	78
*Per3*	5′-TGGTGGTGGTGAATGTAAGAC-3′ 5′-GGCTGTGCTCATCGTTCC-3′	57.2	104
*Cry1*	5′-CAACCTCCATTCATCTTTCC-3′ 5′-CTCATAGCCGACACCTTC-3′	58.9	151
*Cry2*	5′-TGGGCTTCTGGGACTGAG-3′ 5′-GGTAGGTGTGCTGTCTTAGG-3′	57.2	136
*CLOCK*	5′-GCAGCAGCAGCAGCAGAG-3′ 5′-CAGCAGAGAGAATGAGTTGAGTTG-3′	61.9	149
*BmalI*	5′-TGCCACCAATCCATACACAGAAG-3′ 5′-TTCCCTCGGTCACATCCTACG-3′	60.9	123
*CKIɛ*	5′-TCAGCGAGAAGAAGATGTC-3′ 5′-GAAGAGGTTGCGGAAGAG-3′	58.9	149
*β-actin*	5′-CCCATTTATGAGGGCTACGCG-3′ 5′-CGATGAAGGAGGGCTGGAAGA-3′	61.9	313
*GAPDH*	5′-GCGCTGAGTACGTCGTGGAG-3′ 5′-GCTGATGATCTTGAGGCTGTTG-3′	59.8	173
*HBx*	5′-CGTCCTTTGTCTACGTCCCG-3′ 5′-AAGTTGCATGGTGCTGGTGA-3′	59.4	408
